# Identification and Removal of Potential Contaminants in 16S rRNA Gene Sequence Data Sets from Low-Microbial-Biomass Samples: an Example from Mosquito Tissues

**DOI:** 10.1128/mSphere.00506-21

**Published:** 2021-06-16

**Authors:** Sebastián Díaz, Juan S. Escobar, Frank W. Avila

**Affiliations:** a Max Planck Tandem Group in Mosquito Reproductive Biology, Universidad de Antioquia, Medellín, Antioquia, Colombia; b Vidarium—Nutrition, Health, and Wellness Research Center, Grupo Empresarial Nutresa, Medellin, Antioquia, Colombia; Nanjing Normal University

**Keywords:** metabarcoding analysis, bar coding analysis, contamination, gut microbiota, mosquito, reproductive tract microbiota

## Abstract

The bacterial microbiota of the mosquito influences numerous physiological processes of the host. As low-microbial-biomass ecosystems, mosquito tissues are prone to contamination from the laboratory environment and from reagents commonly used to isolate DNA from tissue samples. In this report, we analyzed nine 16S rRNA data sets, including new data obtained by us, to gain insight into the impact of potential contaminating sequences on the composition, diversity, and structure of the mosquito tissue microbial community. Using a clustering-free approach based on the relative abundance of amplicon sequence variants (ASVs) in tissue samples and negative controls, we identified candidate contaminating sequences that sometimes differed from, but were consistent with, results found using established methodologies. Some putative contaminating sequences belong to bacterial taxa previously identified as contaminants that are commonly found in metagenomic studies but that have also been identified as part of the mosquito core microbiota, with putative physiological relevance for the host. Using different relative abundance cutoffs, we show that contaminating sequences have a significant impact on tissue microbiota diversity and structure analysis.

**IMPORTANCE** The study of tissue-associated microbiota from mosquitoes (primarily from the gut) has grown significantly in the last several years. Mosquito tissue samples represent a challenge for researchers given their low microbial biomass and similar taxonomic composition commonly found in the laboratory environment and in molecular reagents. Using new and published data sets that identified mosquito tissue microbiota from gut and reproductive tract tissues (and their respective negative controls), we developed a simple method to identify contamination microbiota. This approach uses an initial taxonomic identification without operational taxonomic unit (OTU) clustering and evaluates the relative abundance of control sample sequences, allowing the identification and removal of purported contaminating sequences in data sets obtained from low-microbial-biomass samples. While it was exemplified with the analysis of tissue microbiota from mosquitos, it can be extended to other data sets dealing with similar technical artifacts.

## INTRODUCTION

Mosquitoes have a resident bacterial microbiota that is fundamental for several physiological processes, such as larval growth, blood digestion, and immune function (1–3), making manipulation of host bacteria a prospective avenue for reducing vector competence (4). For this reason, a multitude of studies have described microbial communities associated with mosquito tissues from relevant medical genera (e.g., *Aedes*, *Anopheles,* and *Culex*), taking advantage of sequencing methods that identify tissue-associated bacteria. Most studies have focused on gut microbiota in different settings (lab-reared or field-collected insects) and in different physiological contexts (e.g., blood-fed or virus- or parasite-infected insects) (reviewed in references 1, 2, and 5). These studies demonstrate the large taxonomic diversity of bacteria present in the mosquito gut, even at the intraspecific level. Most of the identified bacteria belong to the phylum *Proteobacteria* (e.g., Acinetobacter, *Aeromonas*, *Asaia*, *Comamonas*, Enterobacter, Klebsiella, *Pantoea*, Pseudomonas, and *Serratia*, among others) (5).

In recent years, microbiota associated with other tissues has also been described, most notably the salivary gland and reproductive tract microbiota. The bacterial community of the salivary glands is more diverse than that of the gut, although it shares some members of the core gut microbiota, such as Pseudomonas, *Serratia*, and *Elizabethkingia* (6–8). In the reproductive tract, the ovaries and testes have been the most studied tissues (7–9), although the whole reproductive tract has also been sampled (10). Aside from common bacteria described in the other tissues, such as Acinetobacter, Pseudomonas, *Serratia*, *Comamonas*, and *Corynebacterium*, the ovaries are often characterized by the presence of vertically transmitted symbiont bacteria, such as *Wolbachia* in Aedes albopictus and Culex quinquefasciatus and *Asaia* in Anopheles stephensi (11).

Parallel to advances in microbiota identification techniques that have allowed the description of mosquito tissue microbiota in greater detail, interest has grown in understanding and describing the impact of contaminants introduced by molecular reagents, such as microbiota from DNA extraction kits (referred to as the “kitome” [12–14]), PCR master mix (13, 15), and laboratory facilities (14, 16), and technical issues, such as well-to-well contamination (17) and index switching in sequencing platforms (18, 19). Interestingly, many contaminating sequences described in different studies belong to bacteria commonly associated with mosquito tissues, including Acinetobacter, *Chryseobacterium*, Enterobacter, or Pseudomonas (12, 14, 20). This raises a question about the identification of these bacteria in the mosquito tissues: is it an actual presence or signal from undesired contamination?

To counter the effect of contamination, especially when studying low-biomass samples where contaminants can dominate the sampling (20), researchers have proposed precautionary measures in the DNA extraction and sequencing steps, such as randomizing sample types and treatment groups, decontaminating working areas, and sequencing negative controls (e.g., sampling blanks and DNA extraction reagents) and positive controls (e.g., mock communities) (12, 14, 20–22). For the postsequencing analysis, the use of amplicon sequence variants (ASVs), a clustering-free approach for sequence inference, has been shown to be more sensitive for detecting true contamination sequences than classic operational taxonomic unit (OTU) inference (23). Software has been developed to identify contaminating sequences within experimental samples, with some algorithms based on the expectation that contaminating sequences have a higher prevalence in control samples than in experimental samples (24).

Interest in standardizing methodologies has increased in mosquito microbiota research (25, 26), recognizing that as low-biomass samples, mosquito tissues are prone to sequencing artifacts (25). However, no consensus approach has been developed for the identification and removal of possible contaminating sequences from mosquito tissues to date. Of the mosquito tissue microbiota studies that report amplified sequences from negative-control samples (see Table S1 in the supplemental material), only a few have addressed the quantification and reduction of putative contaminating sequences. One reported the complete removal of sequences detected in controls (27) and two others reported the removal of shared OTUs with relative abundances 10 times greater in control samples than tissue samples (28, 29). Unfortunately, the taxonomic identities of the removed OTUs in these studies were not reported.

10.1128/mSphere.00506-21.1 TABLE S1 Published mosquito tissue microbiota articles reviewed from 2011 to 2020. In bold, the articles that included sequences from both tissue samples and negative controls (blank and/or DNA extraction sample[s]). Download Table S1, DOCX file, 0.02 MB. Copyright © 2021 Díaz et al. 2021 Díaz et al. https://creativecommons.org/licenses/by/4.0/ This content is distributed under the terms of the Creative Commons Attribution 4.0 International license.

In this study, we examined published data sets that identified microbiota from low-biomass tissue—gut and reproductive tract tissues—sampled from *Anopheles* and *Aedes* mosquitoes. These data sets were chosen because they used high-throughput sequencing to identify microbiota and uploaded sequences identified from both experimental and negative-control samples to the NCBI’s BioProject or Sequence Read Archive, allowing us to assess how putative contaminating sequences may have affected their results. These previously published data sets utilized tissues from field-collected adult mosquitoes and were processed in different laboratory settings, giving an uncertainty to the generated data that we could not control. Therefore, we complemented the study with newly generated data sets obtained by us from lab-reared specimens to control dissection and DNA extraction protocols and to design the proper negative controls for the analysis. Using each of these data sets, we developed a simple strategy to identify putative contaminating sequences from mosquito tissue samples and examine our results against those identified by Decontam, an established contamination detection method. Finally, we quantified the impact of removing contaminating sequences on microbiota diversity and structure of the individual data sets.

## RESULTS

We analyzed nine data sets of mosquito gut and reproductive tract (RT) tissues (Table 1) using the same pipeline to remove low-quality sequences (i.e., sequences that did not align with the analyzed 16S rRNA region or chimeric and/or nonbacterial sequences). For the curated data sets, we defined ASVs as sequences clustered at 100% identity (see Materials and Methods). To identify potential contaminating sequences in the data sets and to evaluate the effect of removing contaminating sequences on the alpha and beta diversity indicators, we examined unperturbed data sets and each data set where ASVs shared between tissue and negative controls were completely removed. We also modified each data set to remove ASVs in tissue samples that were present at abundances of ≥1, ≥5, or ≥10% in control samples. These thresholds were chosen to represent low, medium, and high abundances of contaminating sequences, respectively, in the negative-control samples.

**TABLE 1:** Information on the examined data sets

Data set	BioProject accession no.	Host species	Sample source	DNA extraction method	16S rRNA region	Tissue	No. of samples	No. Negative controls	Normalization	Reference
Aedes_Gut	PRJNA644640	*Ae. aegypti/Ae. albopictus*	Lab	Phenol-chloroform	V3-V4	Gut	23 (pools)	6	Yes	This study
Aedes_URT	PRJNA644640	*Ae. aegypti/Ae. albopictus*	Lab	Phenol-chloroform	V3-V4	URT	19 (pools)	6	Yes	This study
Aedes_LRT	PRJNA644640	*Ae. aegypti/Ae. albopictus*	Lab	Phenol-chloroform	V3-V4	LRT	24 (pools)	6	Yes	This study
Aegypti_Gut	PRJEB16334	*Ae. aegypti*	Field	DNeasy kit (Qiagen)	V5-V6	Gut	26 (individuals)	5	No	29
Albopictus_Gut	PRJEB6896	*Ae. albopictus*	Field	DNeasy kit (Qiagen)	V5-V6	Gut	33 (individuals)	1	Yes	28
Anopheles1_Gut	PRJNA415615	*An. nuneztovari*/*An. darlingi*	Field	Salt precipitation	V2	Gut	62 (individuals)	2	No	45
Anopheles2_Gut	PRJNA172065	*An. gambiae/An. coluzzii*	Field	DNeasy kit (Qiagen)	V4	Gut	52 (individuals)	1	No	10
Anopheles2_URT	PRJNA172065	*An. gambiae/An. coluzzii*	Field	DNeasy kit (Qiagen)	V4	URT	57 (individuals)	1	No	10
Anopheles2_LRT	PRJNA172065	*An. gambiae/An. coluzzii*	Field	DNeasy kit (Qiagen)	V4	LRT	59 (individuals)	1	No	10

From the ASVs identified in the negative-control samples, we identified 46 that had a relative abundance of ≥1%, the minimal relative abundance threshold to consider in our removal treatments. These ASVs represented 20 bacterial genera (Table 2). Aside from *Chryseobacterium* and *Cloacibacterium* (*Bacteroidetes*), all belonged to the phylum *Proteobacteria*, with Acinetobacter and *Serratia* being the most common (five of the nine data sets). Most ASVs found in control samples had a low relative abundance in the tissue samples (<5%). Eight ASVs had a relative abundance of ≥5%: two Enterobacter ASVs (21.7% and 9.22% average abundance for the three tissues) and one *Serratia* ASV (10.16% in gut tissues) in the Aedes data sets, *Aeromonas* (9.93%), *Pantoea* (8.75%), Acinetobacter (5.31%), and *Serratia* (5.04%) in the Anopheles1_Gut data set, and one Acinetobacter ASV (5.74% and 5.01% in the upper reproductive tract [URT] and lower reproductive tract [LRT], respectively) in the Anopheles2 data set.

**TABLE 2:** ASVs with an overall relative abundance of ≥1% in negative-control samples

Data set	ASV	Abundance^*a*^				Decontam^*b*^	
		Controls	Gut samples	URT samples	LRT samples	Contamination	*P*
Aedes	Enterobacter	40.06	25.89	16.73	22.48	No	
	**Enterobacter**	**19.64**	**13.05**	**6.27**	**8.33**	**Yes**	**0.36**
	***Serratia***	**5.69**	**10.16**	**2.93**	**3.23**	**Yes**	**0.04**
	*Cutibacterium*	1.11	0	0	0	No	

Aegypti	*Serratia*	6.34	0	NA	NA	No	
	*Halomonas*	5.22	0	NA	NA	No	
	*Halomonas*	4.54	0	NA	NA	No	
	*Serratia*	3.79	0	NA	NA	No	
	*Serratia*	2.18	0	NA	NA	No	
	*Serratia*	2.16	0	NA	NA	No	
	*Halomonas*	2.14	0	NA	NA	No	
	*Halomonas*	2.06	0	NA	NA	No	
	*Halomonas*	1.78	0	NA	NA	No	
	*Halomonas*	1.67	0	NA	NA	No	
	*Vibrio*	1.24	0	NA	NA	No	
	*Marinimicrobium*	1.16	0	NA	NA	No	
	*Serratia*	1.15	0	NA	NA	No	
	*Serratia*	1.08	0	NA	NA	No	
	*Halomonas*	1.06	0	NA	NA	No	
	*Halomonas*	1.06	0	NA	NA	No	

Albopictus	**Pseudomonas**	**60.64**	**2.76**	**NA**	**NA**	**Yes**	**0.16**
	***Chryseobacterium***	**4.82**	**2.66**	**NA**	**NA**	**Yes**	**0.47**
	***Janthinobacterium***	**3.24**	**1.72**	**NA**	**NA**	**Yes**	**0.47**
	**Pseudomonas**	**2.89**	**0.25**	**NA**	**NA**	**Yes**	**0.07**
	**Pseudomonas**	**2.07**	**0.07**	**NA**	**NA**	**Yes**	**0.03**
	**Acinetobacter**	**1.53**	**0.03**	**NA**	**NA**	**Yes**	**0.04**
	***Janthinobacterium***	**1.08**	**0.5**	**NA**	**NA**	**Yes**	**0.38**

Anopheles1	*Aeromonas*	17.74	9.93	NA	NA	No	
	*Pantoea*	12.38	8.75	NA	NA	No	
	***Chryseobacterium***	**9.14**	**1.74**	**NA**	**NA**	**Yes**	**0.31**
	**Acinetobacter**	**6.36**	**2.36**	**NA**	**NA**	**Yes**	**0.38**
	Acinetobacter	3.55	5.31	NA	NA	No	
	***Serratia***	**3.53**	**5.04**	**NA**	**NA**	**Yes**	**0.44**
	*Stenotrophomonas*	3.26	1.61	NA	NA	No	
	***Enhydrobacter***	**1.82**	**3.16**	**NA**	**NA**	**Yes**	**0.45**
	*Thorsellia*	1.54	3.06	NA	NA	No	
	*Enhydrobacter*	1.38	0.23	NA	NA	No	
	**Pseudomonas**	**1.36**	**1.4**	**NA**	**NA**	**Yes**	**0.30**
	**Acinetobacter**	**1.17**	**0.11**	**NA**	**NA**	**Yes**	**0.16**

Anopheles2	***Sphingomonas***	**35.85**	**0.01**	**3.80 × 10**^−^**^3^**	**1.21 × 10**^−^**^3^**	**Yes**	**0.02**
	**Acinetobacter**	**21.94**	**4.36**	**5.74**	**5.01**	**Yes**	**0.47**
	*Caulobacter*	19.83	0	0	0	No	
	**Escherichia*-Shigella***	**8.26**	**2.09**	**2.23**	**2.27**	**Yes**	**0.47**
	***Cloacibacterium***	**4.46**	**0.86**	**1.02**	**0.99**	**Yes**	**0.43**
	**Acinetobacter**	**1.67**	**0.14**	**0.11**	**0.14**	**Yes**	**0.14**
	***Diaphorobacter***	**1.08**	**0.18**	**0.22**	**0.21**	**Yes**	**0.30**

a Overall relative abundance for negative-control samples and tissue samples for each data set. NA, not applicable.

b ASVs found as contamination in the Decontam prevalence-based method are listed in bold, with the associated *P* value.

We compared our identified contaminating ASVs against an established contamination identification software, the Decontam package of R, using its prevalence-based method. We evaluated two user-defined classification thresholds for the performed test: P* = 0.1 (Decontam's default threshold) and P* = 0.5. Using the first criterion, resulting candidate ASVs varied from 20 in the Anopheles2 data set to 341 in the Aedes data set (data not shown); of ASVs with a relative abundance of ≥1% (Table 2), only two were identified in the Decontam output: *Serratia* and *Cutibacterium* in the Aedes data set, and *Sphingomonas* in Anopheles2 data set, with only *Serratia* having an abundance of ≥1% in the tissue samples. Using the classification threshold of a P* value of 0.5 increased the total number of candidates ASVs to 26 in the Anopheles2 data set and 419 in the Aedes data set (the complete list is shown in Data Set S1). With both classification thresholds, no candidate sequence was found for the Aegypti data set. The largest difference between the two classification thresholds was an increase in candidate sequences also present in our list of contamination ASVs, including all Albopictus ASVs, all except one in Anopheles2 and half of the ASVs in Aedes and Anopheles1 data sets (Table 2). Interestingly, Decontam did not classify the most abundant ASVs found in the negative-control data sets of Aedes (Enterobacter) and Anopheles1 (*Aeromonas* and *Pantoea*), which were also found in high abundance in the tissue samples, as contamination.

10.1128/mSphere.00506-21.3 DATA SET S1 ASVs identified as contaminants by the prevalence-based method of Decontam. List of ASVs by dataset with their associated probability and *P* value. ASVs with an overall relative abundance of ≥1% in negative-control samples are listed in red, with the taxonomic classification. Download Data Set S1, XLSX file, 0.1 MB. Copyright © 2021 Díaz et al. 2021 Díaz et al. https://creativecommons.org/licenses/by/4.0/ This content is distributed under the terms of the Creative Commons Attribution 4.0 International license.

We quantified the abundance of ASVs shared between negative-control and tissue samples (Table 3). In eight data sets, shared ASVs represented more than 10% of the tissue sample sequences. For gut samples, there were 66.84% in Aedes_Gut, 56.76% in Albopictus_Gut, 56.58% in Anopheles1_Gut, and 11.78% in Anopheles2_Gut. For RT samples, we noted a proportion of shared sequences similar to that in the gut samples from the same data sets: 62.70% and 56.98%, respectively, in Aedes_URT and Aedes_LRT and 10.91% and 10.50% in Anopheles2_URT and Anopheles2_LRT. The Aegypti_Gut data set represents a unique case where shared ASVs were less than 1% of the tissue sampling; prior to singleton removal, 97% of the sequences corresponded to unique sequences exclusive to individual samples in this data set. As expected, increasing the relative abundance threshold (≥1%, ≥5%, or ≥10%) reduced the number of shared ASVs, as well as their presence in tissue samples. For the 10% threshold, contamination abundance in tissue samples was 38.94% (gut), 30.91% (LRT), and 23% (URT) in the Aedes data sets; 5.75% (URT), 5.01% (LRT), and 4.36% (gut) in the Anopheles2 data sets; 18.68% in Anopheles1_Gut; and 2.76% in Albopictus_Gut.

**TABLE 3:** ASV distribution in the negative-control samples^*a*^

Data set	ASVs ≥1%			ASVs ≥5%			ASVs ≥10%			Total ASVs		
	Total	No. shared	% in tissue samples	Total	No. shared	% in tissue samples	Total	No. shared	% in tissue samples	Total	No. shared	% in tissue samples
Aedes_Gut	4	3	49.10	3	3	49.10	2	2	38.94	1,992	138	66.84
Aedes_URT	4	3	25.93	3	3	25.93	2	2	23.00	1,992	75	62.70
Aedes_LRT	4	3	34.04	3	3	34.04	2	2	30.91	1,992	221	56.98
Aegypti_Gut	16	0	0	2	0	0	NA	NA	NA	307	5	0.08
Albopictus_Gut	7	7	7.99	1	1	2.76	1	1	2.76	1,030	307	56.76
Anopheles1_Gut	12	12	42.70	4	4	22.78	2	3	18.68	299	267	56.58
Anopheles2_Gut	7	6	7.63	4	3	6.46	3	2	4.36	26	16	11.78
Anopheles2_URT	7	6	9.33	4	3	7.97	3	2	5.75	26	22	10.91
Anopheles2_LRT	7	6	8.63	4	3	7.28	3	2	5.01	26	18	10.50

a Total ASVs and ASVs with overall relative abundances of ≥1%, ≥5%, and ≥10% in control samples. ASVs shared with tissue samples and their overall relative abundance in the tissue samples are also shown. NA, not applicable.

We next examined how removing sequences present in negative-control samples affected the composition and structure of tissue samples using the following treatments: complete removal of ASVs found in negative controls and removal of ASVs with abundance thresholds of ≥1, ≥5, or ≥10%. Given the differences in targeted 16S rRNA fragments, DNA extraction methods, and the variety of negative controls utilized in each study (Table 1), alpha and beta diversities were not directly comparable across the data sets. Therefore, for each data set, we compared changes in microbial diversity, focusing on the effect of removal treatments compared to nonremoval.

**FIG 1:**
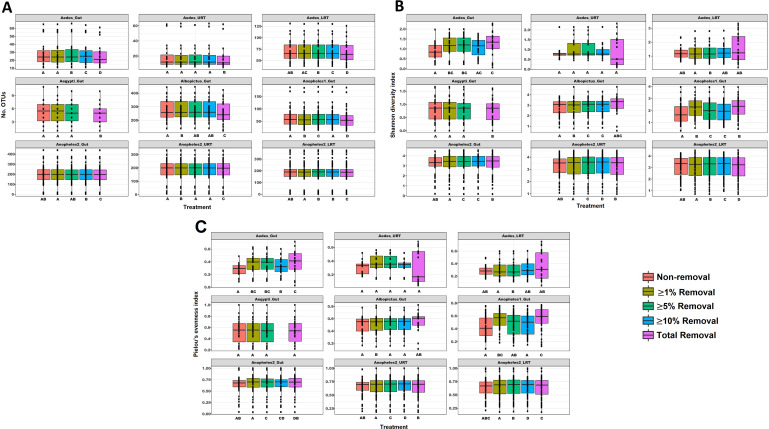
Alpha diversity. (A) Number of OTUs, (B) Shannon diversity index, and (C) Pielou’s evenness index of the unmodified data set or after the removal treatment indicated. Different letters correspond to significant differences at a *P* value of <0.05 for paired *t* tests or Wilcoxon signed-rank tests.

For alpha diversity, in the OTU richness estimator, we found statistically significant differences in the richness for all data sets after total removal of the sequences found in control samples, the most drastic treatment (Fig. 1A). However, we noted that less drastic treatments led to significant differences as well (Table S2), although there were a few exceptions: the Aedes_URT and Aegypti_Gut data sets, where the OTU richness was lower, and two of the three Anopheles2 data sets (gut and LRT), where few ASVs with relative abundances of ≥1% were detected and removed as contamination. For the Shannon index (Fig. 1B), all the data sets except Aedes_URT and Aedes_LRT, at least one removal treatment showed significant differences compared to nonremoval (Table S2). For Pielou’s index (Fig. 1C), we observed less significant differences not only against the nonremoval group but between all treatments, even with two data sets, Aedes_URT and Aegypti_Gut, without any difference between treatments.

10.1128/mSphere.00506-21.2 TABLE S2 *P* values for paired *t* tests or Wilcoxon signed-rank tests. *P* values for comparisons between nonremoval group against removal treatments. Asterisks indicate statistically significant differences. *, *P* = 0.05 to 0.005; **, *P* = 0.0049 to 0.0005; ***, *P* < 0.00049. Download Table S2, DOCX file, 0.02 MB. Copyright © 2021 Díaz et al. 2021 Díaz et al. https://creativecommons.org/licenses/by/4.0/ This content is distributed under the terms of the Creative Commons Attribution 4.0 International license.

**FIG 2:**
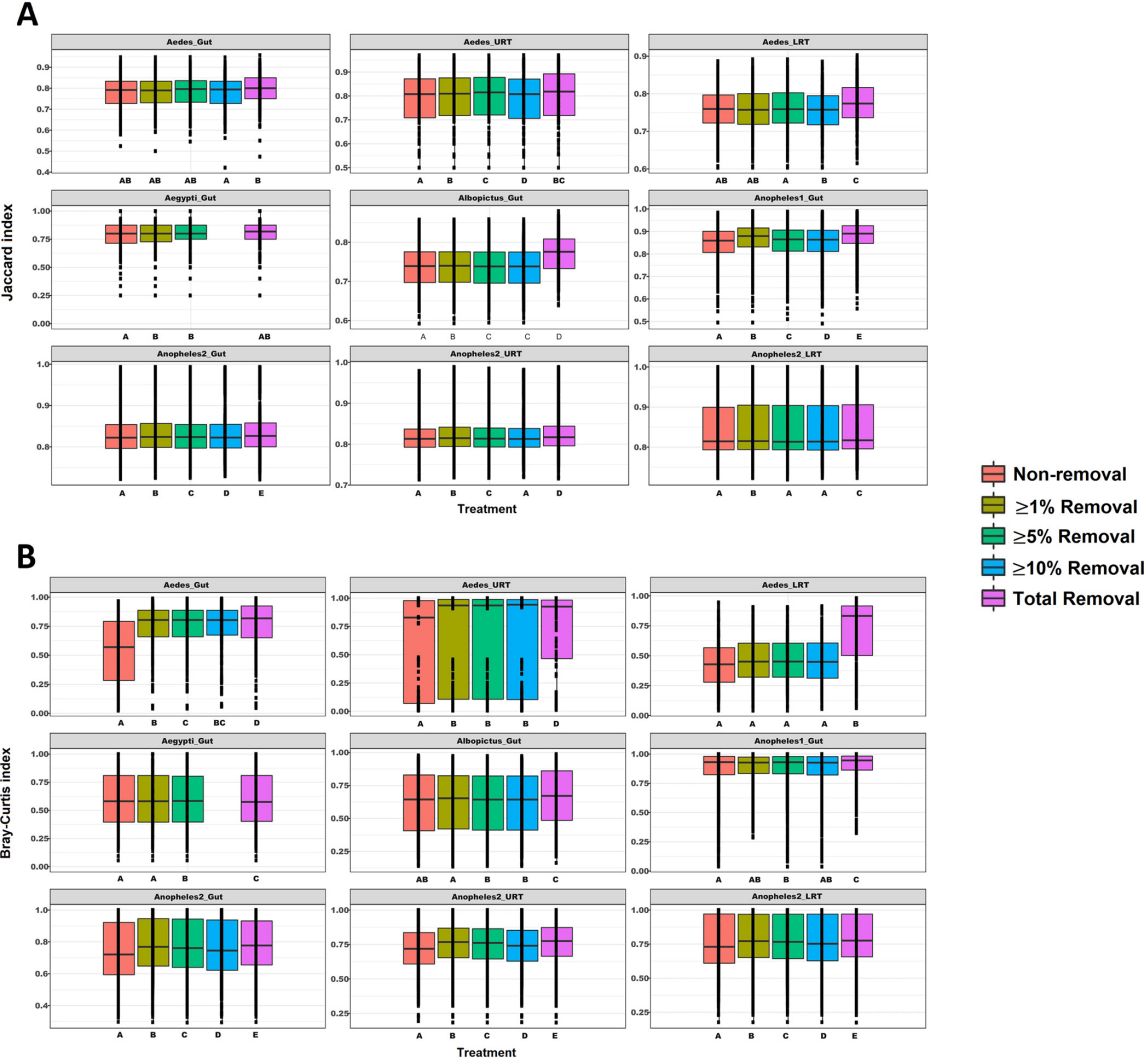
Beta diversity. (A) Jaccard index and (B) Bray-Curtis index of the unmodified data set or after the removal treatment indicated. Different letters correspond to significant differences at a *P* value of <0.05 for paired *t* tests or Wilcoxon signed-rank tests.

For beta diversity, we used the Jaccard (Fig. 2A) and Bray-Curtis (Fig. 2B) indices, finding statistically significant differences between the total removal of the potential contamination OTUs and the nonremoval treatment for all data sets, except in Aegypti_Gut in the Jaccard index (Table S2). Moreover, compared to alpha diversity indices, beta diversity metrics were more sensitive to statistically significant changes using any of the evaluated removal treatments compared to the nonremoval treatment (Table S2).

## DISCUSSION

With our survey of available mosquito tissue data sets and new ones reported here, we highlight the impact of potential contaminants on the composition, structure, and diversity of low-microbial-biomass samples. One remarkable result is that in the data sets of different tissues from the same studies (i.e., Aedes and Anopheles2, where gut, URT, and LRT were sampled), the overall abundance of the contamination sequences was similar between all samples. These results are expected in cases where the sample randomization during DNA extraction and sequencing were successful in avoiding a batch effect.

To identify putative contaminating sequences, we used a clustering-free approach to increase the precision of the removal strategy, which works well for this purpose because contaminants are likely to be specific, as previously demonstrated (23, 30). This puts forward a simple method to trim contaminating sequences that does not depend on taxonomic reference databases. We compared our results against an established method, the prevalence-based identification in Decontam. In this method, the user defines the classification thresholds. In our analysis, we evaluated the default P* value of 0.1 and the more stringent P* value of 0.5. We focused on the comparison of the predicted contaminating sequences that correspond to ASVs with an overall abundance ≥1% (Table 2). We consider the P* value of 0.5 to be more accurate for comparison to our list of candidate ASVs, given that in both cases the abundance of sequences was more important than statistical significance.

In most of the data sets we assessed, we obtained similar results between our proposed method and Decontam with a classification threshold of a P* value of 0.5. In the Albopictus_Gut and Anopheles2 data sets, most negative-control ASVs were classified as contamination. However, there were differences between our method and Decontam: only half of the putative contaminating ASVs we identified in the Aedes_Gut and Anopheles1_Gut data sets and none of the Aegypti_Gut data set (Table 2) were classified as contamination by Decontam. As Decontam bases contamination identification on sequences more prevalent in negative-control than in experimental samples, we suspect that results obtained with this method did not match our candidates in Aedes_Gut data set as the contaminant ASVs were absent in tissue samples, such as *Cutibacterium* and *Caulobacter* in the Aedes and Anopheles2 data sets, respectively. In the Anopheles1_Gut, the candidate ASVs not detected by Decontam included two ASVs with higher abundances in tissue samples compared to controls (Acinetobacter and *Thorsellia*), as well as two ASVs with high abundances in controls and tissue samples (*Aeromonas* and *Pantoea*). Dahlberg et al. (31) found similar results for cow milk microbiota, another low-biomass sample type, where Decontam (using a P* value of = 0.5) could identify some, but not all, of the ASVs with a relative abundance of 1% in the control samples.

The use of a clustering-free approach allows the separation of potential contamination of sequences with the same taxonomy but a different biological origin. In our study, we found abundant ASVs detected in negative controls classified within Acinetobacter, *Chryseobacterium*, Enterobacter, or Pseudomonas, genera previously described as common contaminants (12–14, 20). However, other ASVs classified in these same genera were found in tissue samples but were not detected in negative controls. This illustrates the advantage of a clustering-free approach, as these bacterial groups have been reported as part of the core microbiota in *Aedes* and *Anopheles* mosquitoes (1, 2, 5) with putative functional roles in their hosts. Enterobacter has hemolytic activity associated with blood digestion and egg production (32); Enterobacter and Pseudomonas reduce vector competence for *Plasmodium* infection (33, 34) and La Crosse virus (35); and Acinetobacter and *Chryseobacterium* may contribute to larval development (36).

Distinguishing laboratory or reagent contamination from cross-contamination with experimental samples is particularly challenging in low-microbial-biomass samples, where ASVs with low abundance are ubiquitous. Our analysis showed that most ASVs found in negative controls had low abundance (≤1%). However, mosquito endosymbionts were also detected in low abundance in control samples but in high abundance in tissue samples, suggesting cross-contamination from tissue samples to the negative controls. For instance, *Wolbachia* sequences were found in low abundance (<1%) in control samples of the Aedes, Albopictus_Gut, and Anopheles1 data sets, sequences that can have distinct biological origins (37). *Thorsellia*, another bacterium reported as a natural mosquito symbiont (10, 38, 39), was present in the control sample of the Anopheles2 data set with an abundance of 1.54% (Table 2). Thus, ecological data such as ASVs primarily associated with mosquito tissues or common laboratory contaminants should be considered when interpreting microbial community results (21), as ASVs identified in negative controls may be products of cross-contamination. As our strategy gives results comparable to those of the prevalence-based method, differing only in a subset of identified sequences, and also identifies possible instances of cross-contamination, we suggest that it can be implemented in combination with Decontam with a stringent classification threshold of a P* value of at least 0.5.

In addition to identifying contaminating sequences, we also evaluated trimming strategies to reduce their effects on data analysis. Most removal strategies affected microbial inference, a major result that highlights the impact of ignoring contamination and the crucial role of negative controls to remove potential sources of noise. Our first strategy, removing all the sequences found in negative controls, is considered a very conservative method, where it is preferable to pay the cost of eliminating the true positives than to keep contaminants in the final data set. However, the loss of biological data due to cross-contamination between experimental and the negative-control samples (e.g., well-to-well contamination [17] or index switching [18]), is a reason why some authors discourage this method, suggesting that removal of sequences present in negative controls should be performed only when it can be ensured that they correspond to actual contaminants (22) and propose the use of alternative methods (20).

Another approach assessed for removing contamination sequences was the use of abundance thresholds. Some authors have employed this approach in the study of mosquito-associated microbiota. For instance, Minard et al. (28) removed all shared OTUs with relative abundances at least 10 times greater in control samples than in tissue samples. Our method was based solely on the abundance of the sequences in the negative controls. We observed that microbial inference was severely affected not only by complete removal of sequences present in control samples but also by removal based on abundance thresholds (i.e., ≥1, ≥5, or ≥10% cutoffs). However, we do not consider the total removal of sequences found in negative controls or any predefined abundance threshold as universal to determine contamination. Each study needs to define its proper criteria according to the data obtained from sequencing and the quality of the controls used.

Complementary to the strategy proposed here, it is necessary to establish additional measures to identify and reduce contamination in the analysis of low-microbial-biomass samples (12, 20, 22). These procedures include (i) maximizing the starting sample biomass by choice of sample type, filtration, or enrichment; (ii) randomization of samples and treatments to avoid batch/day effects; (iii) recording batch numbers of reagents; (iv) sequencing of many negative controls that cover all sample processing steps (i.e., dissection, DNA extraction, and library preparation); (v) sequencing of positive controls (e.g., mock community and high-biomass samples with known composition) that can help to detect cross-contamination; and (vi) reporting negative-control sequences in genomic repositories, along with tissue sample sequences.

In summary, our analysis of mosquito tissue microbiota data sets revealed the common presence of contaminant sequences that significantly affected the composition, diversity, and structure of the inferred microbial community. To minimize this impact, we proposed a clustering-free approach to complement the identification of potential contaminants and evaluated different abundance thresholds to gauge the impact of high- and low-abundance ASVs on the inferred microbial community. This strategy should be complemented with laboratory protocols to minimize sample contamination along with the inclusion of as many controls as possible to identify contaminating sequences.

## MATERIAL AND METHODS

### Data acquisition.

We analyzed microbial DNA sequences from mosquito tissue and control samples using two data sources: three newly developed data sets which we report here, and six data sets retrieved from previous studies. The new data sets used the following species: Aedes aegypti collected in Bangkok, Thailand, and maintained in colony since 2009, and Aedes albopictus collected in Medellín, Colombia, and maintained in colony since 2017. For each species, eggs were hatched under vacuum pressure (−50 kPa), and larvae were reared at a density of 200/liter in double-distilled water (ddH_2_O) supplemented with four Hikari Gold cichlid food pellets (Hikari, Himeju, Japan). Pupae were transferred to 5-ml tubes to ensure virginity, and adults were separated into sex-specific cages upon eclosion. Larval rearing and adult maintenance were carried out in an incubator at 27°C and 80% relative humidity. Adults had access to 10% sucrose *ad libitum*. Four- to six-day-old adults were used in our analysis.

We assessed tissues from (i) virgin females, (ii) mated females, and (iii) mated, blood-fed females. Females were individually mated by placing a single pair into an 8-liter container until a copulation occurred, defined as genitalia engagement of ≥10 s for *Ae. aegypti* (40, 41) and ≥30 s for *Ae. albopictus* (42). A subset of females were blood fed on the arm of a volunteer 20 min after mating. Blood feeding on human subjects was approved by the Bioethics Committee of the Sede de Investigación Universitaria (Universidad de Antioquia), and all volunteers signed a consent form. At 24, 48, and 72 h postmating, females were knocked down on ice and stored at −80°C until tissue dissection. The tissues analyzed were gut, upper reproductive tract (URT) (female: ovaries; male: testes), and lower reproductive tract (LRT) (female: oviduct, spermathecae, spermathecal vestibule, and bursa; male: vas deferens, accessory glands, and seminal vesicles), referred to here as Aedes_Gut, Aedes_URT, and Aedes_LRT, respectively. Tissues were dissected in 1× phosphate-buffered saline (PBS) under sterile conditions to obtain pools of 20 tissues per sample stored in sodium chloride-Tris-EDTA (STE) buffer. In total, we sampled 23 gut pools (21 females and 2 males), 19 URT pools (17 females and 2 males), and 24 LRT pools (22 females and 2 males).

Samples were lysed by adding 6 μl of lysozyme (20 μg/μl) for 2 h at 37°C, followed by an overnight incubation at 56°C upon addition of 24 μl of proteinase K (20 μg/μl). DNA was extracted using a phenol-chloroform protocol and resuspended in 50 μl of AE buffer (Qiagen, Valencia, CA, USA). Experimental samples and a PBS sterile blank control were randomly seeded in five different extraction rounds, each with a DNA extraction control. Positive samples for a diagnostic 16S rRNA gene PCR using primers P338F and 1492R and negative controls were sent to Macrogen (Seoul, South Korea) for sequencing on the Miseq Illumina platform using the primers Bakt_341F (5′-CCT ACG GGN GGC WGC AG-3′) and Bakt_805R (5′-GAC TAC HVG GGT ATC TAA TCC-3′) (43), which amplified the V3-V4 hypervariable regions of 16S rRNA gene with an average sequencing depth of 100,000 reads per sample.

To identify published reports of mosquito tissue microbiota, we used PubMed (https://pubmed.ncbi.nlm.nih.gov/) to search for articles with title words [Mosquito] (OR [*Aedes*] OR [*Anopheles*] OR [*Culex*]) AND [Microbiota], complementing this effort with a more extensive manual search. We focused on studies that used high-throughput gene sequencing with data made available at the NCBI’s BioProject or Sequence Read Archive (SRA). We found 24 articles from 2011 to 2020 (Table S1) that matched our criteria. From this group, we selected five studies with available sequences from both gut samples and negative controls (blank and/or DNA extraction sample[s]), and one including gut, URT, and LRT samples for male and female mosquitoes. One gut data set was discarded given the limited number of sequences in control samples (44). The remaining data sets used in our analysis were from gut samples of *Ae. aegypti* (referred to as Aegypti_Gut) (29), *Ae. albopictus* (Albopictus_Gut) (28), and Anopheles darlingi*/*Anopheles nuneztovari (Anopheles1_Gut) (45) and from the gut and reproductive tract tissue of Anopheles gambiae*/*Anopheles coluzzii (Anopheles2_Gut, Anopheles2_URT, and Anopheles2_LRT) (10) (Table 1).

### Microbiota analyses.

For the nine data sets analyzed, raw reads were processed following the standard operating procedure (SOP) for MiSeq sequences of Mothur v. 1.43.0 (46). Low-quality sequences were filtered out according to established parameters: (i) presence of ambiguous nucleotides, (ii) sequences with more than 8 homopolymers, (iii) sequence length lower than the 2.5% percentile, and (iv) sequence length higher than the 97.5% percentile. The remaining sequences were preclustered to reduce sequencing errors (allowing one difference every 100 bp), and chimeras were removed with VSEARCH (47), as well as nonbacterial sequences, based on a preliminary classification using the SILVA v132 database (48). Singletons were removed from the final data set. For the Aedes (Aedes_Gut, Aedes_URT, and Aedes_LRT) and Albopictus_Gut data sets, data were normalized to 25,000 sequences per sample because of their high sequencing depth.

To evaluate the effect of removing contaminating sequences on microbial composition and diversity, we conducted the following analyses. First, we used a clustering-free approach to identify independent bacterial subpopulations shared by tissue samples and negative controls. Each unique sequence (i.e., 100% nucleotide identity) was defined as an amplicon sequence variant (ASV). Subsequently, we evaluated different removal treatments of potential contamination. We did not remove sequences based on abundance comparisons between control and experimental samples, as has been previously explored for mosquito tissue data sets (28, 29), but instead focused exclusively on the relative ASV abundance in the control samples. Five new subsets were created: (i) the original data set with no removal of ASVs found in negative controls (a common approach in mosquito microbiome studies); (ii) a data set removing all ASVs present in control samples, also implemented in some mosquito studies (27); (iii) a data set with a minimal threshold, removing ASVs with an overall relative abundance of ≥1% in control samples; (iv) a data set with an intermediate threshold, removing ASVs with an overall relative abundance of ≥5% in control samples; and (v) a data set with the biggest threshold, removing ASVs with an overall relative abundance of ≥10% in control samples. Finally, for each of the above subsets, we clustered sequences at 97% sequence identity to obtain standard OTUs using the OptiClust algorithm implemented in Mothur (49).

Our methodology was based on considering as potential contamination any ASV present in negative-control samples. To corroborate our results against other published methods, we used the Decontam R package (24) to identify contamination ASVs. Decontam uses two strategies to identify contaminating sequences higher in frequency in low-concentration samples (frequency based) and/or found in negative controls (prevalence based). As concentration data are not available for the published data sets, we used the prevalence-based method, which performs a chi-square test for each sequence assuming that the likelihood of detecting a contaminating sequence will be higher in control samples than in experimental samples. For each ASV, the test creates an associated static *P* score that is compared to a user-defined classification threshold (P*). If *P* is less than P*, the sequence is classified as a potential contaminant. We performed the analysis using two approaches: one using the default classification threshold of a P* value of 0.1, and the more stringent approach of using a P* value of 0.5. There is no consensus on which approach is better. Instead, a recommended practice is to run an additional threshold together with the default and to compare the results obtained (24, 50, 51).

To evaluate the changes in microbiota composition, diversity, and structure upon sequence removal, we calculated alpha and beta diversity indices for each removal treatment in each data set. Specifically, we calculated the number of OTUs as a measure of microbial richness, Shannon diversity index, and Pielou’s evenness index. We also calculated the Jaccard and Bray-Curtis indices as measures of beta diversity. After evaluating normality of the indices for each subset using a Shapiro-Wilk test, we used paired *t* tests and Wilcoxon signed-rank tests to determine whether there was a statistically significant difference between alpha and beta diversity indices in the subsets where putative contaminants were removed compared to the original, unaltered data sets. To increase the statistical power of these tests, we combined all samples from each distinct data set even if they differed in species of origin (Aedes, Anopheles1, and Anopheles2 data sets), reproductive status (mated or virgin in the Aedes data set), and nutritional status (blood fed or not blood fed in the Aedes and Anopheles1 data sets), as we expected that all samples from a particular study would be prone to similar sources of contamination, since they originated from the same laboratory environment and were processed with the same molecular reagents.

### Data availability.

New data sets are available in the NCBI Sequence Read Archive (SRA) repository, under BioProject accession code PRJNA644640.
