# Supplementary material for: Identification and Removal of Potential Contaminants in 16S rRNA Gene Sequence Data Sets from Low-Microbial-Biomass Samples: an Example from Mosquito Tissues
Source: mSphere. 2021 Jun 16;6(3):e00506-21. doi: 10.1128/mSphere.00506-21 (PMC8265668; doi:10.1128/mSphere.00506-21)
Supplement: TABLE S1 [file msphere.00506-21-st001.docx]

| **Year** | **Title** | **Tissue evaluated** | **Bioproject/SRA accesion number** | **Samples available** | **Controls avalaible** | **DOI** |
| --- | --- | --- | --- | --- | --- | --- |
| 2011 | Dynamic Gut Microbiome across Life History of the Malaria Mosquito *Anopheles gambiae* in Kenya | Gut | SRA031282.2 | Yes | No | [doi.org/10.1371/journal.pone.0024767](https://doi.org/10.1371/journal.pone.0024767) |
| 2012 | Deep sequencing reveals extensive variation in the gut microbiota of wild mosquitoes from Kenya | Gut | SRR516961 | Yes | No | [doi.org/10.1111/j.1365-294X.2012.05759.x](https://doi.org/10.1111/j.1365-294X.2012.05759.x) |
| 2012 | Midgut Microbiota of the Malaria Mosquito Vector *Anopheles gambiae* and Interactions with *Plasmodium falciparum* Infection | Gut | SRS281724.1-25.1 | Yes | No | [doi.org/10.1371/journal.ppat.1002742](https://doi.org/10.1371/journal.ppat.1002742) |
| 2014 | Salivary glands harbor more diverse microbial communities than gut in *Anopheles culicifacies* | Gut/SG | SRX368316 | Yes | No | [doi.org/10.1186/1756-3305-7-235](https://doi.org/10.1186/1756-3305-7-235) |
| 2015 | Developmental succession of the microbiome of *Culex* mosquitoes | Gut | PRJEB6788 | Yes | No | doi.org/10.1186/s12866-015-0475-8 |
| 2015 | Antibiotics in ingested human blood affect the mosquito microbiota and capacity to transmit malaria | Gut | PRJEB7708 | Yes | No | [doi.org/10.1038/ncomms6921](https://doi.org/10.1038/ncomms6921) |
| **2015** | **French invasive Asian tiger mosquito populations harbor reduced bacterial microbiota and genetic diversity compared to Vietnamese autochthonous relatives** | Gut | **PRJEB6896** | **Yes** | **Yes** | [doi.org/10.3389/fmicb.2015.00970](https://doi.org/10.3389/fmicb.2015.00970) |
| 2016 | Dynamics of Bacterial Community Composition in the Malaria Mosquito's Epithelia | Gut/URT/SG | SRR1038490-93 | Yes | No | [doi.org/10.3389/fmicb.2015.01500](https://doi.org/10.3389/fmicb.2015.01500) |
| 2016 | Effects of environment, dietary regime and ageing on the dengue vector microbiota: evidence of a core microbiota throughout *Aedes aegypti* lifespan | Gut | SRR2916651 | Yes | No | [doi.org/10.1590/0074-02760160238](https://doi.org/10.1590/0074-02760160238) |
| 2016 | Mosquitoes host communities of bacteria that are essential for development but vary greatly between local habitats | Gut | PRJNA342829 | Yes | No | [doi.org/10.1111/mec.13877](https://doi.org/10.1111/mec.13877) |
| **2016** | **The reproductive tracts of two malaria vectors are populated by a core microbiome and by gender and swarm-enriched microbial biomarkers** | Gut/URT/LRT | **PRJNA172065** | **Yes** | **Yes** | [doi.org/10.1038/srep24207](https://doi.org/10.1038/srep24207) |
| **2017** | **Carryover effects of larval exposure to different environmental bacteria drive adult trait variation in a mosquito vector** | Gut | **PRJEB16334** | **Yes** | **Yes** | [doi.org/10.1126/sciadv.1700585](https://doi.org/10.1126/sciadv.1700585) |
| 2017 | Changes in the microbiota cause genetically modified *Anopheles* to spread in a population | Gut/URT | PRJNA397763/SRP115247 | Yes | No | [doi.org/10.1126/science.aak9691](https://doi.org/10.1126/science.aak9691) |
| 2017 | Comparative analysis of gut microbiota of mosquito communities in central Illinois | Gut | PRJNA374733 | Yes | No | [doi.org/10.1371/journal.pntd.0005377](https://doi.org/10.1371/journal.pntd.0005377) |
| 2018 | Diverse laboratory colonies of *Aedes aegypti* harbor the same adult midgut bacterial microbiome | Gut | PRJEB22905 | Yes | No | [doi.org/10.1186/s13071-018-2780-1](https://doi.org/10.1186/s13071-018-2780-1) |
| 2018 | Estimating bacteria diversity in different organs of nine species of mosquito by next generation sequencing | Gut/URT/SG | PRJNA416453 | Yes | No | [doi.org/10.1186/s12866-018-1266-9](https://doi.org/10.1186/s12866-018-1266-9) |
| **2018** | **Factors shaping the gut bacterial community assembly in two main Colombian malaria vectors** | Gut | **PRJNA415615** | **Yes** | **Yes** | [doi.org/10.1186/s40168-018-0528-y](https://doi.org/10.1186/s40168-018-0528-y) |
| **2018** | **Short-term impacts of anthropogenic stressors on *Aedes albopictus* mosquito vector microbiota** | Gut | **PRJEB25905** | **Yes** | **Yes** | [doi.org/10.1093/femsec/fiy188](https://doi.org/10.1093/femsec/fiy188) |
| 2019 | Bacterial communities associated with the midgut microbiota of wild *Anopheles gambiae* complex in Burkina Faso | Gut | PRJNA558839 | Yes | No | [doi.org/10.1007/s11033-019-05121-x](https://doi.org/10.1007/s11033-019-05121-x) |
| 2019 | Host blood-meal source has a strong impact on gut microbiota of *Aedes aegypti* | Gut | PRJNA494958 | Yes | No | [doi.org/10.1093/femsec/fiy213](https://doi.org/10.1093/femsec/fiy213) |
| 2020 | Altered Gut Microbiota and Immunity Defines *Plasmodium vivax* Survival in *Anopheles stephensi* | Gut | SAMN10496496/SAMN10439711 | Yes | No | [doi.org/10.3389/fimmu.2020.00609](https://doi.org/10.3389/fimmu.2020.00609) |
| 2020 | Comparative Analysis of the Bacterial and Fungal Communities in the Gut and the Crop of *Aedes albopictus* Mosquitoes: A Preliminary Study | Gut | PRJEB23948 | Yes | No | [doi.org/10.3390/pathogens9080628](https://doi.org/10.3390/pathogens9080628) |
| 2020 | Effect of life stage and pesticide exposure on the gut microbiota of *Aedes albopictus* and *Culex pipiens* L | Gut | PRJNA626593 | Yes | No | [doi.org/10.1038/s41598-020-66452-5](https://doi.org/10.1038/s41598-020-66452-5) |
| 2020 | The environment and species affect gut bacteria composition in laboratory co-cultured *Anopheles gambiae* and *Aedes albopictus* mosquitoes | Gut | PRJEB28193 | Yes | No | [doi.org/10.1038/s41598-020-60075-6](https://doi.org/10.1038/s41598-020-60075-6) |
