# Supplementary material for: Identification and Removal of Potential Contaminants in 16S rRNA Gene Sequence Data Sets from Low-Microbial-Biomass Samples: an Example from Mosquito Tissues
Source: mSphere. 2021 Jun 16;6(3):e00506-21. doi: 10.1128/mSphere.00506-21 (PMC8265668; doi:10.1128/mSphere.00506-21)
Supplement: TABLE S2 [file msphere.00506-21-st002.docx]

| **Dataset Name** | **No. OTUs** | | | | **Shannon** | | | | **Pielou** | | | |
| --- | --- | --- | --- | --- | --- | --- | --- | --- | --- | --- | --- | --- |
|  | 1% | 5% | 10% | Total | 1% | 5% | 10% | Total | 1% | 5% | 10% | Total |
| **Aedes_Gut** | 0.2986 | 0.04771 (*) | 0.03666 (*) | 0.0001283 (***) | 0.01166 (*) | 0.008714 (*) | 0.0594 | 0.001686 (**) | 0.007479 (*) | 0.006863(*) | 0.04646 (*) | 0.0007932 (**) |
| **Aedes_URT** | 0.4237 | 0.7728 | 0.7728 | 0.001517 (**) | 0.8971 | 0.8971 | 0.7548 | 0.6507 | 0.9382 | 0.8971 | 0.7983 | 0.2753 |
| **Aedes_LRT** | 0.1703 | 0.3277 | 0.01092(*) | 4.039e-07 (***) | 0.9491 | 0.9452 | 0.6547 | 0.2799 | 0.9485 | 0.9569 | 0.6535 | 0.259 |
| **Aegypti_Gut** | 0.3269 | 1 | NA | 0.004272 (**) | 0.3269 | 0.5307 | NA | 0.0118(*) | 0.3269 | 0.2617 | NA | 0.5311 |
| **Albopictus_Gut** | 0.01759(*) | 2.215e-06 (***) | 2.215e-06 (***) | < 2.2e-16 (***) | 0.01369 (*) | 0.002916 (**) | 0.002916 (**) | 0.3043 | 0.01041 (*) | 0.2491 | 0.2491 | 0.2641 |
| **Anopheles1_Gut** | 1.537e-07 (***) | 0.001551 (**) | 0.965 | 2.778e-11 (***) | 2.206e-06 (***) | 0.008966 (*) | 0.02276 (*) | 1.806e-06 (***) | 0.0001666 (***) | 0.7432 | 0.8405 | 2.436e-05 (***) |
| **Anopheles2_Gut** | 0.7565 | 0.322 | 0.082 | 5.562e-07 (***) | 0.1726 | 0.008647 (*) | 0.0002264 (***) | 0.4635 | 0.0672 | 0.005686(**) | 0.0001283 (***) | 0.1503 |
| **Anopheles2_URT** | 0.01704 (*) | 0.7306 | 0.3876 | 6.073e-08 (***) | 0.2036 | 0.004128 (**) | 5.885e-05 (***) | 0.8488 | 0.08047 | 0.002163(**) | 1.301e-05 (***) | 0.4269 |
| **Anopheles2_LRT** | 0.05037 | 0.6644 | 0.2367 | 2.356e-08 (***) | 0.7045 | 0.2834 | 0.0004153 (***) | 0.03737 (**) | 0.4433 | 0.4092 | 0.00237 (*) | 0.07208 |
| **Dataset Name** | **Jaccard** | | | | **Bray-Curtis** | | | |  |  |  |  |
|  | **1%** | **5%** | **10%** | **Total** | **1%** | **5%** | **10%** | **Total** |  |  |  |  |
| **Aedes_Gut** | 0.007245 (*) | 3.562e-08 (***) | 0.3613 | 1.175e-15 (***) | < 2.2e-16 (***) | < 2.2e-16 (***) | < 2.2e-16 (***) | < 2.2e-16 (***) |  |  |  |  |
| **Aedes_URT** | 0.000921 (**) | 1.065e-08 (***) | 6.384e-05 (***) | 0.0006423 (**) | 0.002346 (*) | 0.002346 (*) | 0.001543 (**) | 1.294e-05 (***) |  |  |  |  |
| **Aedes_LRT** | 0.8838 | 4.279e-09 (***) | 6.364e-15 (***) | < 2.2e-16 (***) | 9.601e-06 (***) | 8.974e-06 (***) | 8.004e-07 (***) | < 2.2e-16 (***) |  |  |  |  |
| **Aegypti_Gut** | 8.752e-06 (***) | 0.03255 (*) | NA | 0.3221 | 1 | 0.005025 (*) | NA | 5.485e-09 (***) |  |  |  |  |
| **Albopictus_Gut** | 0.0178 (*) | < 2.2e-16 (***) | < 2.2e-16 (***) | < 2.2e-16 (***) | 1.587e-12 (***) | 1.06e-07(***) | 1.06e-07 (***) | < 2.2e-16 (***) |  |  |  |  |
| **Anopheles1_Gut** | < 2.2e-16 (***) | < 2.2e-16 (***) | < 2.2e-16 (***) | < 2.2e-16 (***) | 0.6402 | 0.0001513 (***) | 1.031e-13 (***) | < 2.2e-16 (***) |  |  |  |  |
| **Anopheles2_Gut** | < 2.2e-16 (***) | < 2.2e-16 (***) | < 2.2e-16 (***) | < 2.2e-16 (***) | < 2.2e-16 (***) | < 2.2e-16 (***) | < 2.2e-16 (***) | < 2.2e-16 (***) |  |  |  |  |
| **Anopheles2_URT** | < 2.2e-16 (***) | < 2.2e-16 (***) | 1.265e-12 (***) | < 2.2e-16 (***) | < 2.2e-16 (***) | < 2.2e-16 (***) | < 2.2e-16 (***) | < 2.2e-16 (***) |  |  |  |  |
| **Anopheles2_LRT** | < 2.2e-16 (***) | 1.181e-12 (***) | 0.9598 | < 2.2e-16 (***) | < 2.2e-16 (***) | < 2.2e-16 (***) | < 2.2e-16 (***) | < 2.2e-16 (***) |  |  |  |  |
